# Integrated single-cell and bulk transcriptomic profiling reveals cancer-associated fibroblast heterogeneity in glioblastoma and establishes a clinically actionable prognostic model and preliminary experimental validation

**DOI:** 10.1186/s41065-025-00548-8

**Published:** 2025-08-26

**Authors:** Wenhua Zhang, Yaxiong Li, Conghui Li, Qiang Huang

**Affiliations:** 1 Department of Neurosurgery, Tianjin Medical University General Hospital, Tianjin, 300000 China; 2 Department of Neurosurgery, the First Hospital of Hebei Medical University, Shijiazhuang, 050000 Hebei China

**Keywords:** Cancer-Associated fibroblasts, Glioblastoma, Immunotherapy, Nomogram

## Abstract

Cancer-associated fibroblasts (CAFs) critically regulate tumor progression, angiogenesis, metastasis, and therapeutic resistance. This study investigated the characteristics of CAFs in glioblastoma (GBM) and developed a CAF-based risk signature to predict patient prognosis. The single-cell RNA sequencing (scRNA-seq) data were sourced from the Gene Expression Omnibus (GEO) database, whereas the bulk RNA-seq datasets were retrieved from The Cancer Genome Atlas (TCGA) and Chinese Glioma Genome Atlas (CGGA), respectively. The Seurat R package processed scRNA-seq data to identify CAF clusters using established markers. Prognostic genes were screened through univariate Cox regression, with Lasso regression constructing the final risk model. A nomogram incorporating clinical parameters was subsequently developed. Immunohistochemical validation was performed using the Human Protein Atlas (HPA) for the signature genes. The qRT-PCR validation was conducted in U251 and HA cells. ScRNA-seq analysis revealed five CAF clusters in GBM, including three prognostically relevant subtypes. Three key genes were refined to construct a risk signature functionally enriched in the the IL6_JAK_STAT3 signaling, P53 pathway, and inflammatory response. The signature correlated strongly with stromal and immune scores. Multivariate analysis confirmed risk signature independent prognostic value (*P* < 0.0001), followed by age (*P* = 0.005). The CAF-derived nomogram demonstrated superior predictive accuracy for 1-/2-year survival compared to clinical factors alone. The signature genes were validated in the HPA database and experimental validation. This study proposes CAF-derived molecular signatures as potential predictors of glioblastoma prognosis worthy of clinical validation. Systematic characterization of CAF heterogeneity may offer insights for interpreting GBM immunotherapy responses, providing a foundation for future exploration of stroma-targeted therapeutic strategies.

**Supplementary Information:**

The online version contains supplementary material available at 10.1186/s41065-025-00548-8.

## Introduction

Glioma represents an aggressive primary brain tumor with dismal prognosis, where GBM demonstrates the highest malignancy grade, comprising > 50% of glioma cases. Notably, ~ 70% of low-grade glioma patients progress to secondary GBM within 5–10 years [1]. Despite therapeutic advances, median survival post-diagnosis remains < 24 months, coupled with a 5-year survival rate of merely 5.6% [2, 3], largely attributable to inevitable recurrence. Recent multi-omics advancements have substantially enhanced our understanding of GBM pathogenesis, facilitating the development of molecular signatures for outcome prediction. Nevertheless, novel multigene signatures are urgently needed to refine prognostic stratification and recurrence monitoring.

The tumor microenvironment (TME) encompasses neoplastic cells and stromal components. While tumor aggressiveness was historically attributed solely to malignant cells [4], emerging evidence highlights critical cancer-stromal crosstalk in disease progression [5]. Stromal constituents comprise fibroblasts, pericytes, mesenchymal stem cells, and immune populations embedded within extracellular matrix proteins [6]. Cancer-associated fibroblasts (CAFs) – derived from mesenchymal stem cells, endothelial cells [4, 7], adipocytes, or even transformed tumor cells [8] – constitute pivotal TME regulators. Prevalent in breast, prostate, and hepatocellular carcinomas [9, 10], CAFs drive tumor progression through growth factor/cytokine secretion, extracellular matrix remodeling, and therapy resistance induction [11–14], even in cancer cell-free contexts [15]. Consequently, targeting CAF- mediated protumorigenic signaling has emerged as a promising therapeutic strategy for GBM.

Despite growing CAF research in GBM, systematic characterization of CAF heterogeneity and its clinical/immunotherapeutic implications remains limited. Leveraging scRNA-seq and bulk transcriptomic data from public repositories, we identified CAF subclusters and constructed a CAF-derived prognostic signature. We further elucidated its clinical relevance, immune landscape associations, and immunotherapy response patterns. To facilitate clinical translation, we developed a CAF-integrated nomogram incorporating standard clinicopathological parameters. This work provides mechanistic insights into CAF-mediated TME modulation, potentially guiding personalized therapeutic approaches for GBM management.

## Materials and methods

### Data acquisition and processing

Single-cell RNA sequencing data (accession GSE162631) comprising 4 glioblastoma tumor core and 4 matched peripheral tissue samples were obtained from the Gene Expression Omnibus (GEO). Single-cell data underwent quality filtering using the following criteria: genes detected in ≥ 3 cells and cells containing ≥ 250 expressed genes. Mitochondrial and ribosomal RNA content was quantified using Seurat’s PercentageFeatureSet function: mitochondrial proportion = 15%. Subsequent filtering retained cells with ≥ 6,000 expressed genes and unique molecular identifiers (UMIs) > 100, yielding 24,597 high-quality cells. Bulk RNA-seq data, somatic variant profiles (single-nucleotide variants [SNVs] and copy number alterations [CNVs]), and clinical metadata for GBM were retrieved from TCGA. Samples lacking survival information were excluded, resulting in 153 tumor specimens (Table 1). The CGGA_693 cohort (*n* = 249) served as an independent validation dataset after excluding non-tumor specimens and cases with incomplete follow-up data. Ten oncogenic pathways (Cell Cycle, HIPPO, MYC, NOTCH, NRF1, PI3K, TGF-β, RAS, TP53, WNT) were analyzed as previously described [16].

**Table 1:** Clinicopathological characteristics of glioma patients in the cancer genome atlas (TCGA-GBM) discovery cohort

Characteristics		No. of Patients
**Age**		
≤ 60		77
>60		76
**Gender**		
Male		99
Female		54
**Grade**		
Grade IV		153
**Subtypes**		
Proneural		18
Neural		5
Classical		48
Mesenchymal		66
NA		16
**IDH mutation**		
Mutation		10
Wildtype		139
NA		4
**1p/19qcodeletion**		
Non-codeletion		147
NA		6
**MGMT methylation**		
Methylation		52
Unmethylation		70
NA		31

### Definition of CAF

ScRNA-seq data reanalysis was performed using Seurat [17]. Initial quality control excluded cells with < 250 or > 6,000 expressed genes, followed by log-normalization. Batch correction between two samples was achieved via Harmony. Cell clustering was performed using the FindNeighbors/FindClusters algorithm (dims = 40, resolution = 0.6) following Uniform Manifold Approximation and Projection (UMAP)-based dimensionality reduction (20 principal components, resolution = 0.6). t-SNE visualization was implemented via RunTSNE. Fibroblast identification employed canonical markers (*ACTA2*, *FAP*, *PDGFRB*, *NOTCH3*). Subclustering reused the FindNeighbors/FindClusters pipeline, with subsequent t-SNE visualization. Cluster-specific markers were identified using FindAllMarkers (log [Fold Change/ FC] ≥ 0.5, min.pct = 0.35, adjusted *P* < 0.05). KEGG pathway analysis of marker genes utilized clusterProfiler [18], while CopyKAT [19] assessed CNV patterns to distinguish malignant from non-malignant cells.

### Identification of hub genes of CAF

Differentially expressed genes (DEGs) between tumor and normal tissues were identified using the limma package (FDR < 0.05, |log₂FC|>1) [20]. CAF cluster-correlated genes were selected via Pearson analysis (*P* < 0.001, correlation coefficient > 0.4). Prognosis-associated genes were subsequently screened through univariate Cox regression (*P* < 0.05, survival R package v2.42-3). A multivariate Cox model-derived risk signature was constructed using the formula: Risk score = Σ(β_i × Exp_i) where β_i represents coefficient values and Exp_i denotes normalized gene expression. Patients were categorized into high- and low-risk groups through z-score standardization. Predictive performance was evaluated by time-dependent ROC analysis (timeROC package) (https://cran.r-project.org/web/packages/timeROC/index.html). Validation cohort analyses followed identical protocols.

### Immune microenvironment profiling

The CIBERSORT algorithm [21]quantified 22 immune cell subtypes in TCGA-GBM samples. Stromal/immune scores were computed via ESTIMATE to characterize tumor microenvironment composition.

### Prognostic model development

Clinicopathological variables and risk scores underwent univariate/multivariate Cox regression (significance threshold: *P* < 0.05). Significant multivariate predictors were integrated into a nomogram using the rms package [22]. Model calibration was evaluated using calibration curves and decision curve analysis (DCA).

### Immunotherapy response prediction

Transcriptomic data from anti-PD-1-treated TCGA-GBM cases were analyzed to evaluate the risk signature’s predictive value for immune checkpoint blockade (ICB) response.

### Analysis of expression levels of signature genes

To investigate differential expression patterns of the four signature genes between normal cerebral cortex and glioma tissues, we performed a reanalysis of raw mRNA sequencing data from the GTEx and TCGA databases. Parallel evaluation of protein expression levels was conducted through immunohistochemistry analysis using representative tissue specimens from the Human Protein Atlas (HPA) database.

### Cell culture

The commercial human glioblastoma cell lines U251MG and HA were sourced from the American Type Culture Collection (ATCC) through Xiamen Yimo Biotechnology Co., Ltd. All cell lines were authenticated using STR profiling and tested for mycoplasma contamination before experiments. The cells were cultured in DMEM (Beyotime, China) with 10% fetal bovine serum (FBS; Beyotime, China) at 37 °C in a 5% CO₂ environment.

### Quantitative RT-PCR

Total RNA was extracted using TRIzol™ (Invitrogen, USA). qRT-PCR was performed with AceQ SYBR Green Master Mix (Q121-02, Jizhen Biotechnology, China). Genes expression was calculated via the 2 − ΔΔCt method. Primer sequences: LITAF: F 5′-ATGTCGGTTCCAGGACCTTAC-3′, R: 5′-TACGAAGGAGGATTCATGCCC-3′; OMR: F 5′-TCGTGGAGCCCTTCG-3′, 5′-GTTCAGCCAAGACTTCACTC-3′; TCF12: F 5′-TCTGCCCCTAGATGAGACCT-3′, R 5′-GGCAATCATTCGGTCCTGTC-3′; β-actin: F 5′-GTGAAGGTGACAGCAGTCGGTT-3′, R 5′-GAAGTGGGGTGGCTTTTAGGA-3′. Reactions were performed in technical triplicates across three biological replicates.

### Statistical methods

Analyses utilized R software v3.6.3. Correlation matrices employed Pearson/Spearman methods. Group comparisons used Wilcoxon tests. Survival differences were assessed via Kaplan-Meier analysis with log-rank testing. Statistical significance was defined as *P* < 0.05.

## Results

### CAF identification in scRNA-seq data

**Fig. 1:**
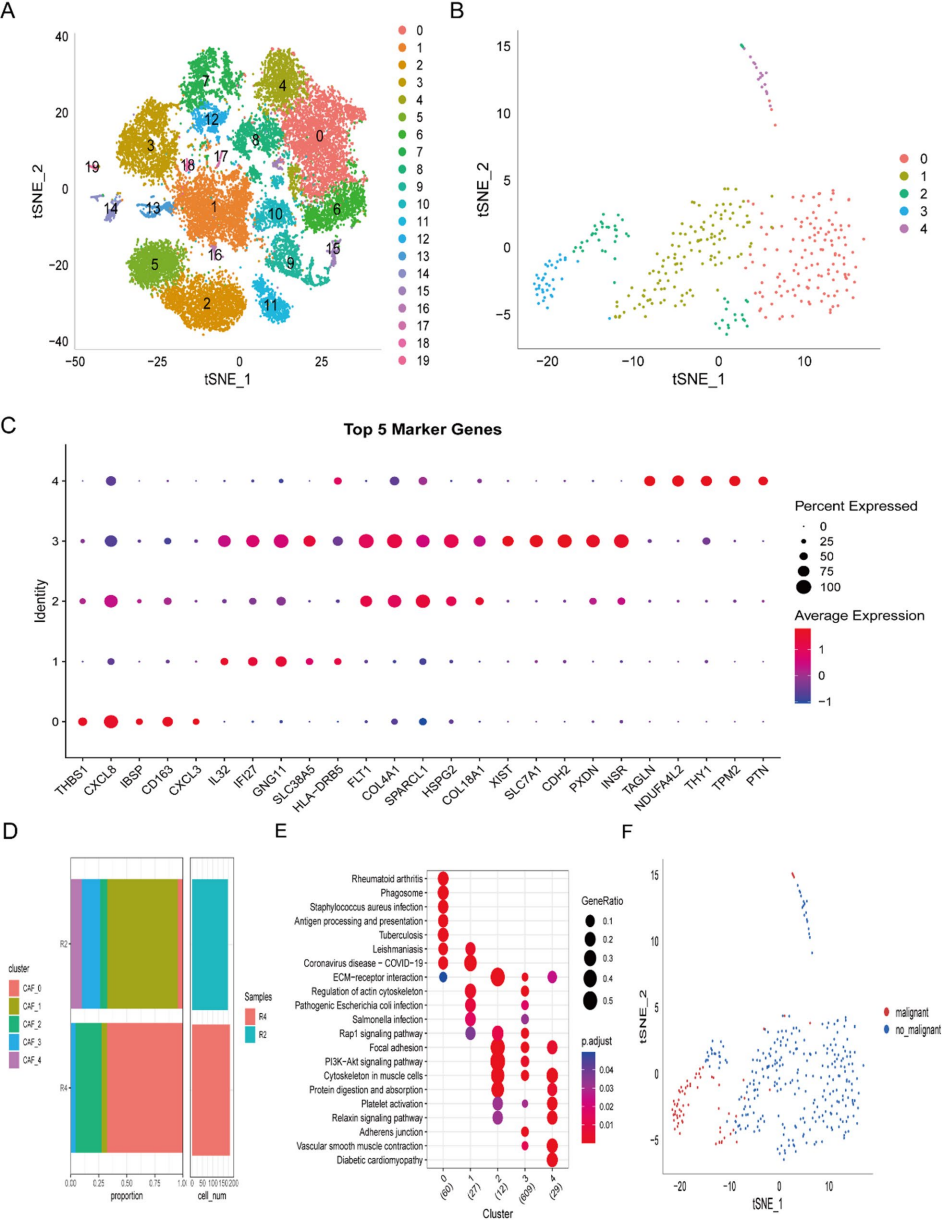
Identification of cancer-associated fibroblast (CAF) clusters in glioblastoma (GBM) scRNA-seq datasets. (**A**) t-SNE projection illustrating sample distribution (*n* = 20); (**B**) t-SNE visualization of fibroblast clustering outcomes (5 subpopulations); (**C**) Dot plot demonstrating subgroup-specific expression of top 5 marker genes; (**D**) Comparative analysis of CAF subgroups in tumor vs. adjacent tissues: cellular proportion and population size; (**E**) KEGG pathway enrichment analysis across five fibroblast subpopulations; (F) t-SNE mapping of CopyKat-predicted malignant/non-malignant cell distribution

Initial processing yielded 24,597 cells (Fig. S1). Following log-normalization and non-linear projection, eight cellular subpopulations emerged. Five CAF populations were delineated through canonical marker expression (*ACTA2*, *FAP*, *PDGFRB*, *NOTCH3*) (Fig. S2A, S2B). Cells from five CAFs clusters were extracted for further clustering and dimensionality reduction. The same clustering algorithm was then applied, identifying five distinct CAFs clusters (Fig. S2C, S2D). t-SNE visualization indicated that the GBM microenvironment comprised 5 biologically distinct subpopulations (mesCAF, iCAF, myCAF, apCAF, unclassified) (Fig.S3). None of the five CAFs subpopulations showed expression of the epithelial cell-specific gene, thereby validating the reliability of CAFs detection (Fig.S4).t-SNE visualization confirmed 20 samples distribution patterns (Fig. 1), with final classification identifying five CAF clusters for downstream analysis (Fig. 1B). Comparative analysis revealed 1,452 inter-cluster DEGs, with heatmap visualization highlighting cluster-specific top markers (Fig. 1C). Cohort-specific CAF cluster distributions are shown in Fig. 1D. KEGG pathway analysis demonstrated significant enrichment in Antigen processing and presentation, ECM-receptor interaction, Vascular smooth muscle contraction, Cytoskeleton in muscle cells, PI3K-AKT signaling, and focal adhesion (Fig. 1E). Copy number variation profiling classified 354 cells as malignant versus non-malignant across clusters (Fig. 1F).

### Pathway activation in CAF subpopulations

**Fig. 2:**
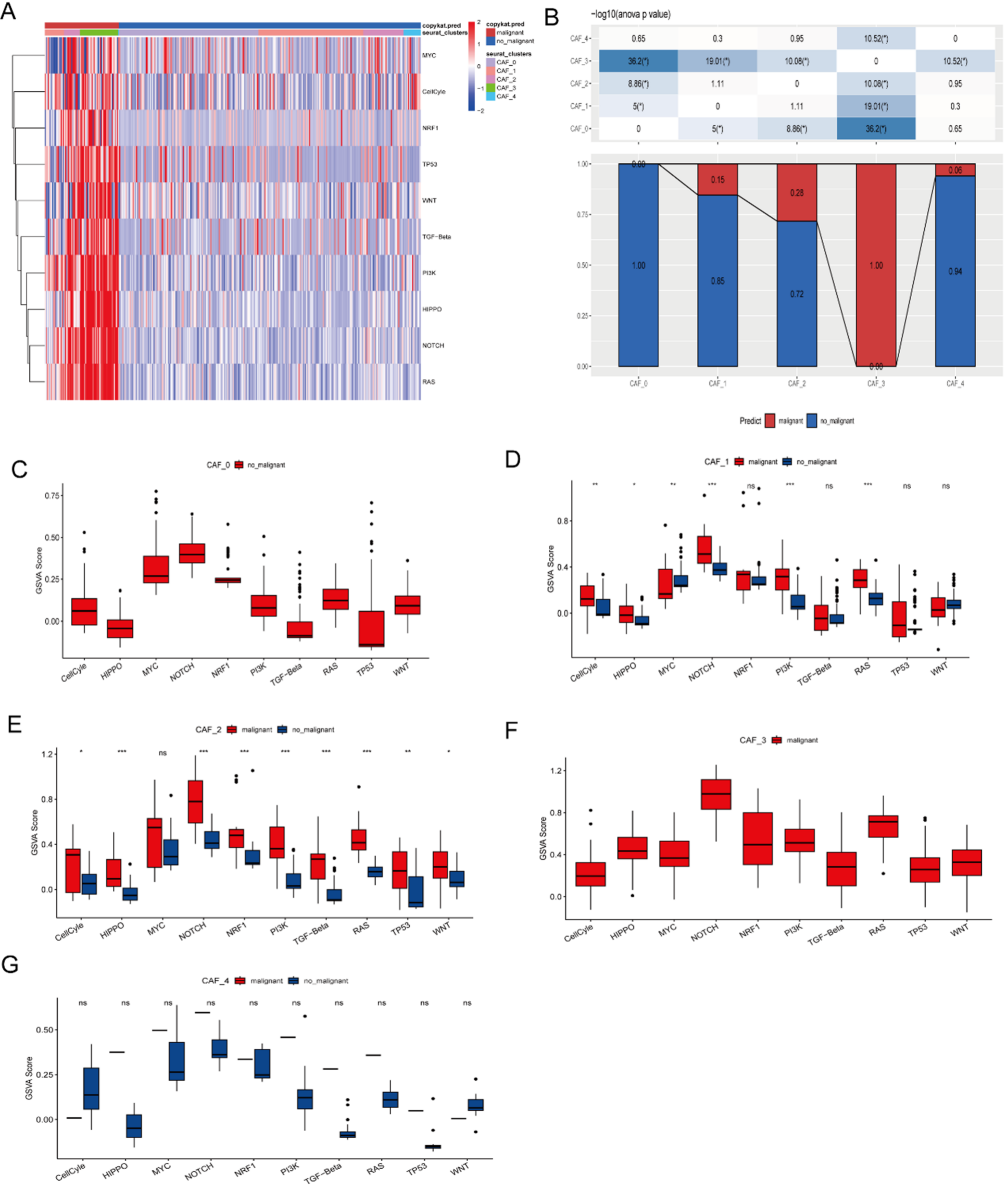
Pathway characterization of cancer-associated fibroblast clusters. (**A**) Heatmap of tumor-associated pathway enrichment scores across CAF subpopulations (top 10 pathways shown), (**B**) Comparative analysis of CAF clusters between malignant and non-malignant cells; GSVA score comparisons for individual pathways in malignant vs. non-malignant cells: CAF_0 (**C**), CAF_1 (**D**), CAF_2 (**E**), CAF_3(**F**), and CAF_4(**G**). (Wilcoxon test; *P < 0.05, **P < 0.01, ***P < 0.001, ****P < 0.0001; ns, not significant)

**Fig. 3:**
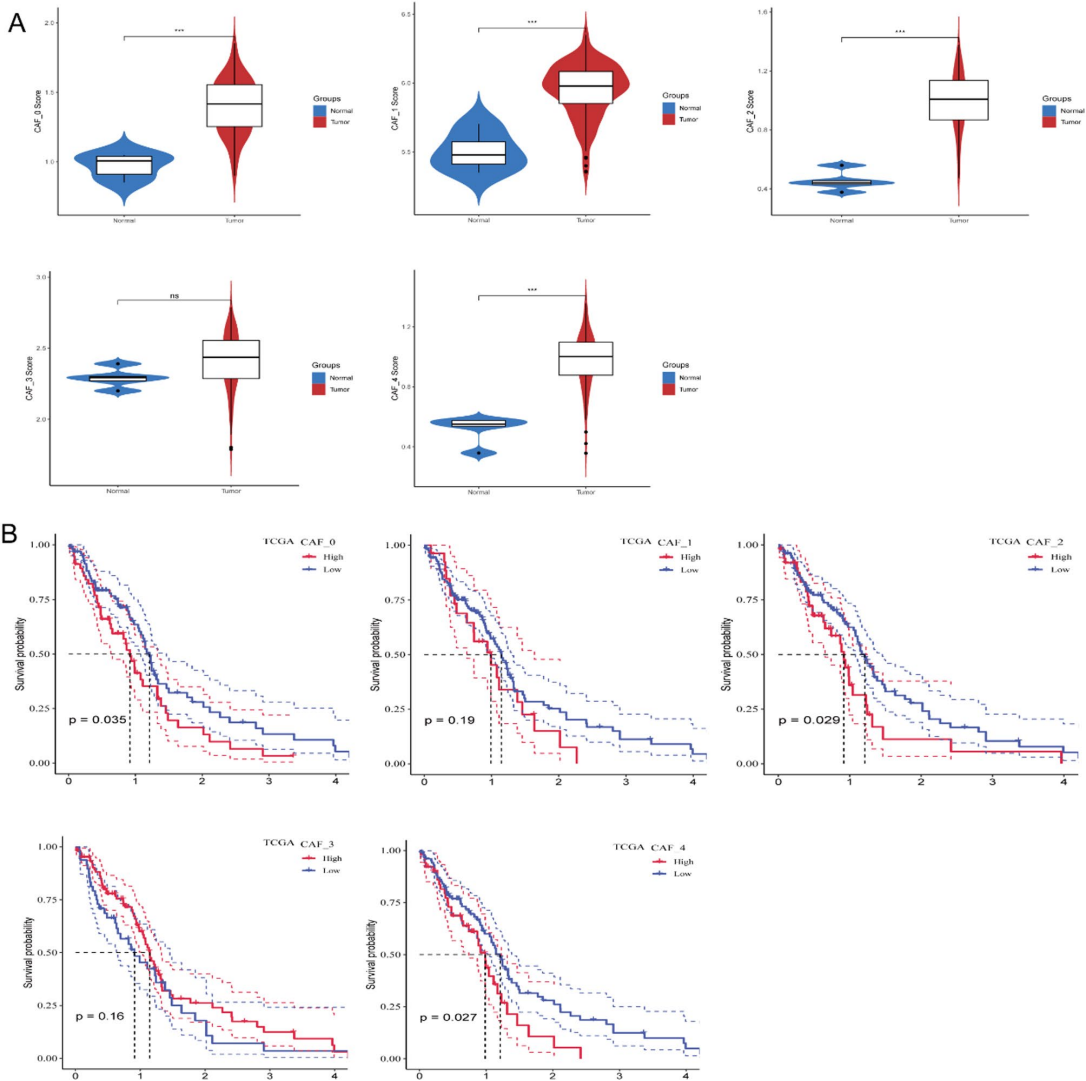
Associations among the four CAF clusters and prognosis in GBM patients. (**A**) Comparative analysis of CAF scores across four clusters in tumor versus normal tissues. (**B**) Kaplan-Meier (K-M) survival curves comparing patients with high versus low CAF scores within the five clusters. (**P < 0.01; ***P < 0.001; ****P < 0.0001)

Functional associations between cancer-associated fibroblast (CAF) clusters and tumor progression were investigated, we analyzed ten oncogenic pathways across five CAF subsets. GSVA scores revealed distinct pathway activation patterns among clusters (Fig. 2A). CAF_3 exhibited significantly higher malignant cell proportions versus other clusters (Fig. 2B). Additionally, we examined the GSVA scores of ten tumor-related pathways in both malignant and non-malignant cells across each CAF cluster, noting minor differences (Fig. 2C-G). To assess the links between CAF clusters and prognosis, we first calculated the ssGSEA score for the marker genes (the top 5 DEGs of CAF clusters identified in Fig. 1C) in each CAF cluster using the TCGA cohort. The data indicates that tumor samples had significantly higher scores in the CAF_0, CAF_1, CAF_2, and CAF_4 clusters compared to normal samples (Fig. 3A). Survival stratification revealed poorer outcomes for high-CAF_0, CAF_2, and CAF_4 groups, while CAF_1/CAF_3 clusters exhibited limited prognostic relevance (*P* > 0.05) (Fig. 3B). These findings suggest CAF_0, CAF_2, and CAF_4 drive progression despite CAF_1/CAF_3 enrichment in GBM.

### CAF-Associated hub gene identification

**Fig. 4:**
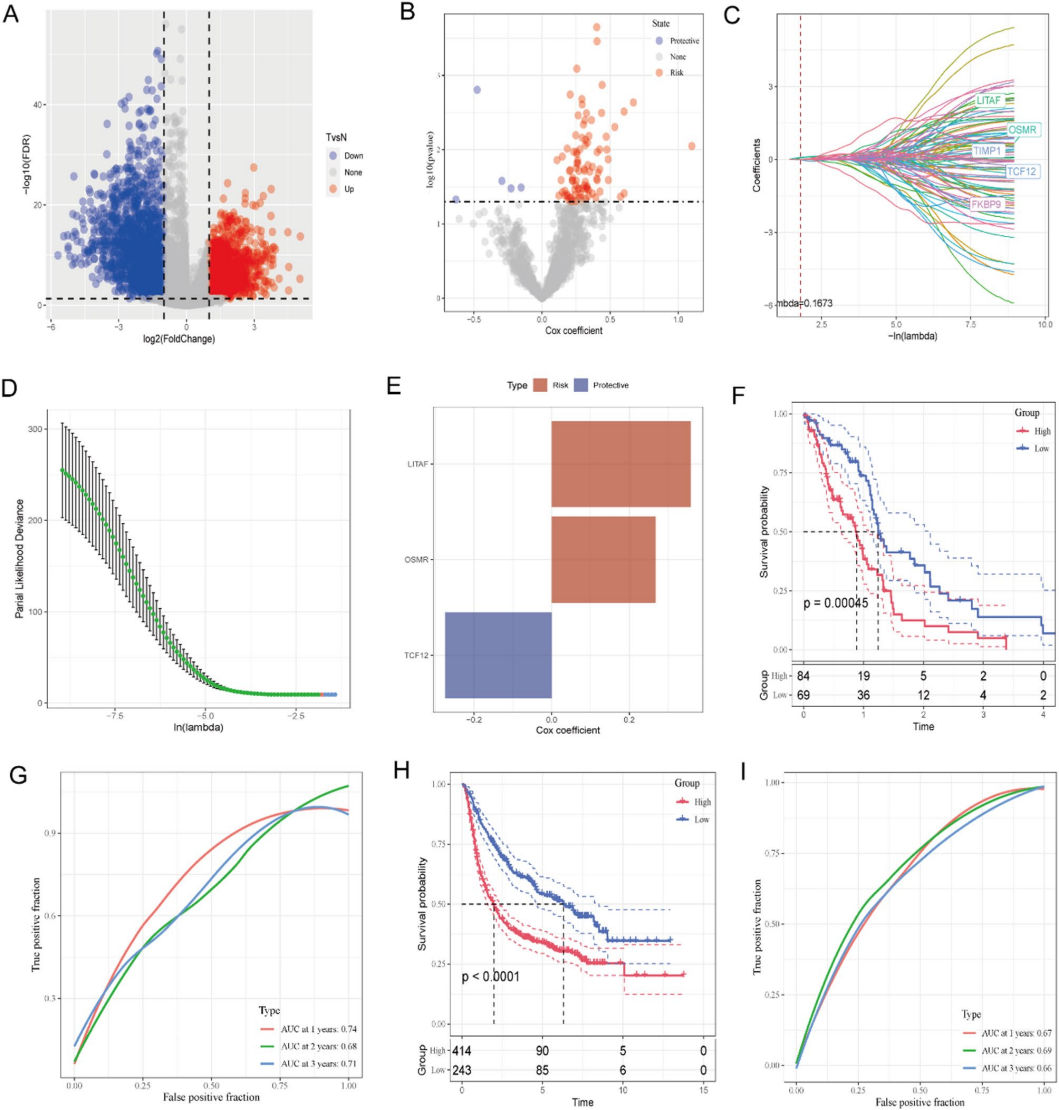
Identification of prognostic signature hub genes. (**A**) Volcano plot of differentially expressed genes (DEGs) between tumor and normal tissues (TCGA cohort). (**B**) Prognosis-associated DEGs identified by univariate Cox regression. (**C**) LASSO coefficient trajectory across λ values. (**D**) Coefficient distributions during λ-parameter optimization. (**E**) Multivariate Cox regression coefficients for signature genes. (**F-G**) TCGA cohort validation: Kaplan-Meier survival analysis and ROC curves for the 3-gene model. (**H-I**) CGGA cohort validation: Corresponding survival and ROC analyses. (**P < 0.01; ***P < 0.001; ****P < 0.0001)

Differentially expressed genes (DEGs) in tumor versus normal tissues comprised 2,032 upregulated and 1,857 downregulated transcripts. (Fig. 4A). Among 3,889 DEGs, 1069 showed significant correlations with prognostic CAF clusters. Univariate Cox regression identified 98 survival-associated genes (Fig. 4B). Lasso regression (λ = 0.1673) refined the panel to 5 candidates (Fig. 4C, D), with multivariate analysis ultimately selecting three genes: Lipopolysaccharide-Induced TNF-α Factor (*LITAF*), Onconstatin M receptor (*OSMR*), Transcription factor 12 (*TCF12*) (Fig. 4E). The risk score formula: Risk Score = 0.358×*LITAF* + 0.268×*OSMR* + (-0.274) ×*TCF12.* Z-score normalization stratified patients into high-/low-risk groups. The model demonstrated robust predictive capacity in TCGA (1-year AUC = 0.74, 3-year AUC = 0.71) and CGGA_693 cohorts (1-year AUC = 0.67, 3-year AUC = 0.66) (Fig. 4F, H). Kaplan-Meier analysis confirmed significantly poorer survival in high-risk groups across both cohorts (log-rank *P* < 0.001) (Fig. 4G, I).

### Genetic alterations and pathway associations

**Fig. 5:**
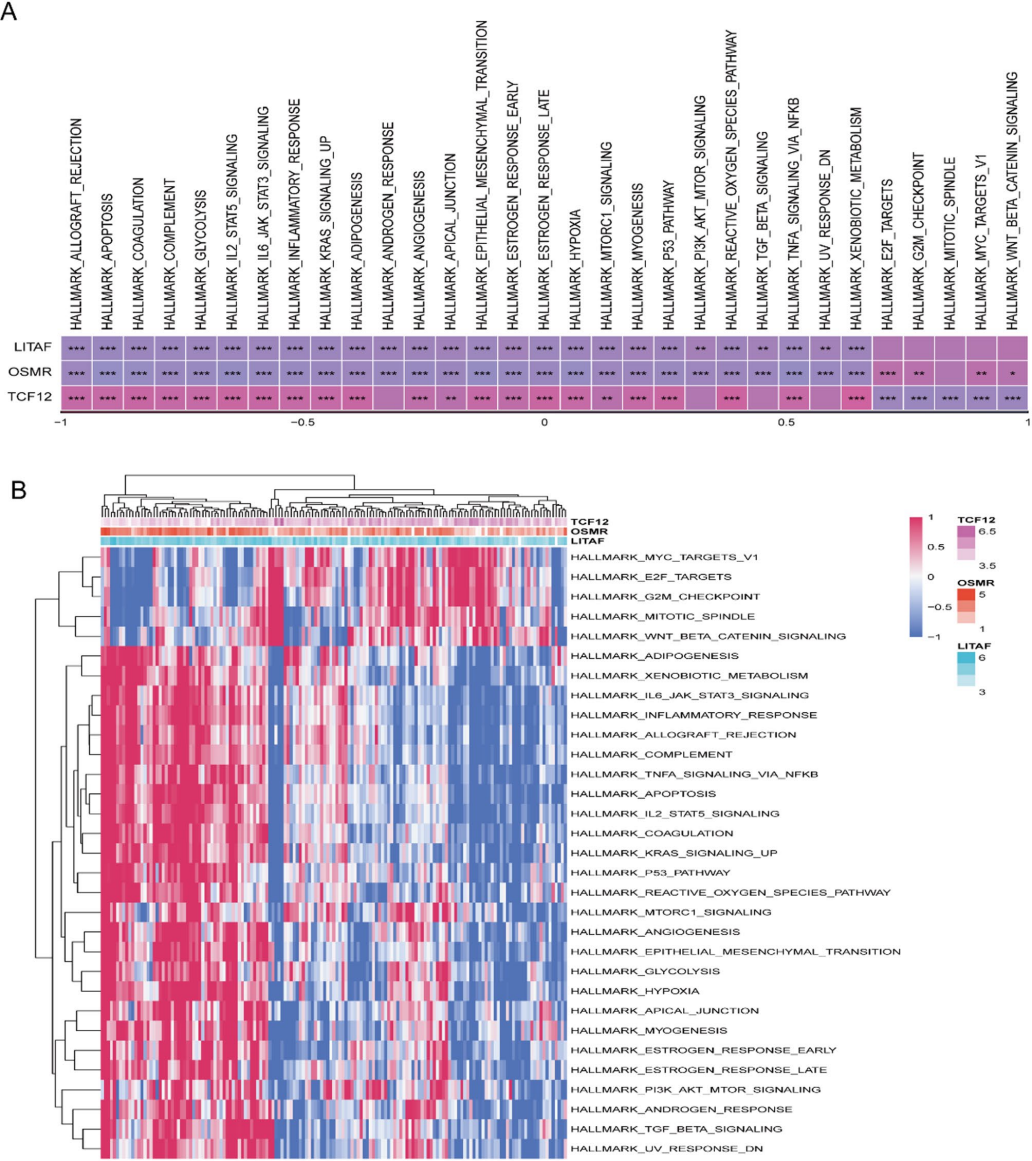
Functional pathway analysis of risk-associated genes. (**A**) Heatmap of gene-pathway correlation analysis. (**B**) Heatmap of enrichment scores for key pathways. (*P < 0.05; **P < 0.01; ***P < 0.001)

The next step was to analyze the SNV mutations in each of the three genes used to develop the risk signature. Analysis of signature genes revealed recurrent SNVs in *TCF12* and *OSMR*, while *LITAF* remained wild-type across cohorts (Fig. S5A). Mutation co-occurrence analysis demonstrated significant associations between *OSMR* and established oncogenic driver (*TP53*; *P* < 0.05) (Fig. S5B). CNVs were infrequent across all signature genes (Fig. S5C). Pathway enrichment analysis mapped the three genes to 31 oncogenic processes, prominently featuring the IL6_JAK_STAT3 signaling, P53 pathway, Inflammatory response, and Apoptosis (Fig. 5A, B).

### Immune microenvironment correlations

Gene expression analysis revealed distinct immunomodulatory associations: *LITAF* and *OSMR* exhibited positive correlations with stromal, immune, and ESTIMATE scores. *TCF12* showed inverse relationships (Fig.S6A). Median-stratified analysis confirmed elevated stromal/immune scores in high-expressing *LITAF* and *OSMR* groups versus low-expressing counterparts (*P*>0.005;Fig. S6B). Immune cell correlation profiling identified: *TCF12* inversely associated with Neutrophils cells, Monocytes cells, T_cells_CD8 cells and T_cells_CD4_memory_activated; *LITAF* positively correlated with Macrophages_M2, T_cells_CD8 cells, and neutrophils cells; *OSMR* positively correlated with Natural Killer _cells _resting and T_cells_CD4_memory_activated (Fig. S6C). Analysis of correlations revealed that LITAF and OSMR showed a strong positive correlation with fibroblasts, while TCF12 demonstrated a strong negative correlation with fibroblasts (Fig. S6D).

### Association of risk score with PD-L1 Blockade immunotherapy response

**Fig. 6:**
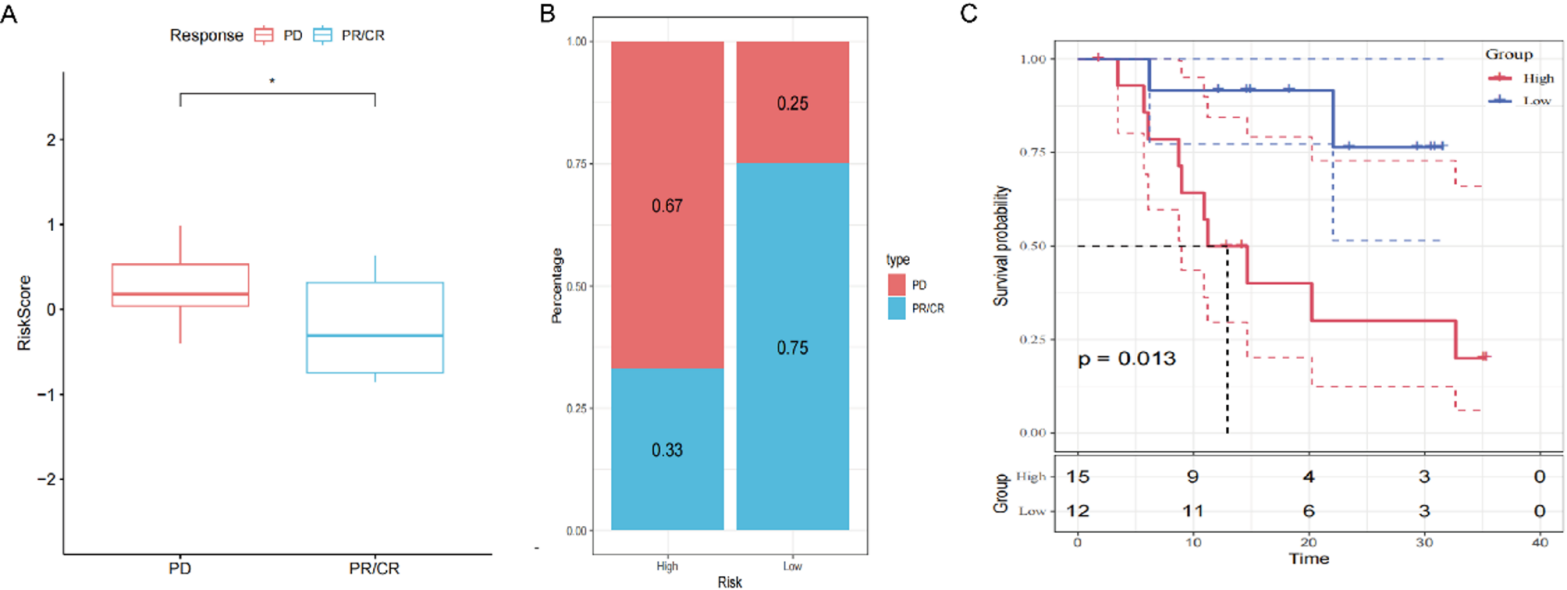
Association of risk score with PD-L1 blockade immunotherapy response. (**A**) Differential risk scores across immunotherapy response categories in the TCGA cohort. (**B**) Distribution of immunotherapy responses stratified by risk score groups (TCGA cohort). (**C**) Prognostic stratification by risk score groups in the TCGA cohort. (*P < 0.05)

T-cell immunotherapy, an anticancer strategy demonstrating synergistic survival benefits [23], was evaluated for prognostic relevance to immune checkpoint inhibition using the TCGA-GBM cohort. To evaluate the prognostic utility of our risk signature for immune checkpoint inhibition, we analyzed the TCGA-GBM cohort. Anti-PD-L1 treated patients in the TCGA-GBM cohort exhibited differential response patterns: complete response (CR), partial response (PR), and progressive disease (PD). Comparative analysis revealed significantly elevated risk scores in PD patients versus CR/PR subgroups (Fig. 6A). High-risk patients demonstrated greater PD prevalence compared to low-risk counterparts (Fig. 6B). Survival analysis confirmed superior clinical outcomes for low-risk patients, showing prolonged overall survival versus high-risk subjects in TCGA-GBM cohorts (*P* = 0.013; Fig. 6C).

### Identification of independent prognostic factors and nomogram construction

**Fig. 7:**
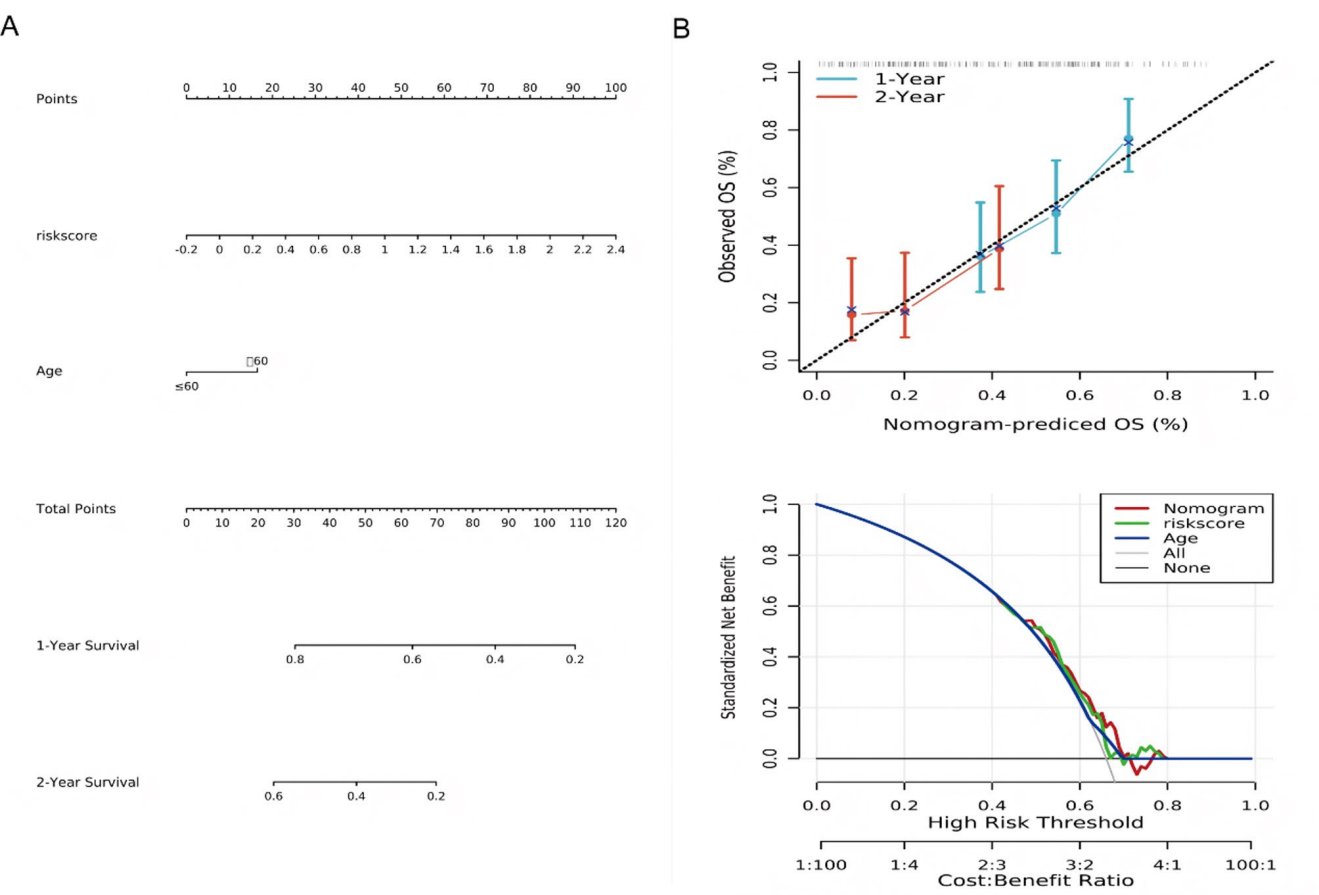
Prognostic nomogram construction for glioblastoma. (**A**) Age-integrated nomogram for survival prediction. (**B**) 1-/2-year calibration plot accuracy and clinical net benefit assessment via decision curve analysis (DCA)

To enhance the predictive capacity of the risk model, we performed univariate and multivariate Cox regression analyses incorporating clinicopathological parameters and risk scores. Multivariate analysis identified the risk score as the most significant independent predictor of glioblastoma outcomes (HR = 1.51, 95% CI: 1.12–2.03, *P* < 0.0001), with age demonstrating secondary prognostic value (HR = 2.04, 95% CI: 1.24–3.35, *P* = 0.005) (Table 2). Consequently, we developed a nomogram incorporating age and risk score (Fig. 7A). The calibration curves showed excellent agreement between predicted and observed survival probabilities and decision curve analysis further confirmed the superior clinical utility of the integrated nomogram for risk stratification compared to individual parameters (Fig. 7B).

**Table 2:** Univariate/multivariate Cox regression of risk scores and clinical parametersl in TCGA-GBM cohorts

Characteristics	Univariate Cox regression			Multivariate Cox regression	
	Hazard ratio (95% CI)	*P* value		Hazard ratio (95% CI)	*P* value
Age	2.34(1.46–3.76)	< 0.0001		2.04(1.24–3.35)	0.005
Gender	0.87(0.55–1.38)	0.548		0.97(0.57–1.63)	0.895
IDH status: WT vs. Mut	6.84(1.66–28.24)	0.008		1.96(0.38–10.05)	0.420
risk score	1.66(1.30–2.12)	< 0.0001		1.51(1.12–2.03)	< 0.0001

### Validation of signature genes’ expression profiles

**Fig. 8:**
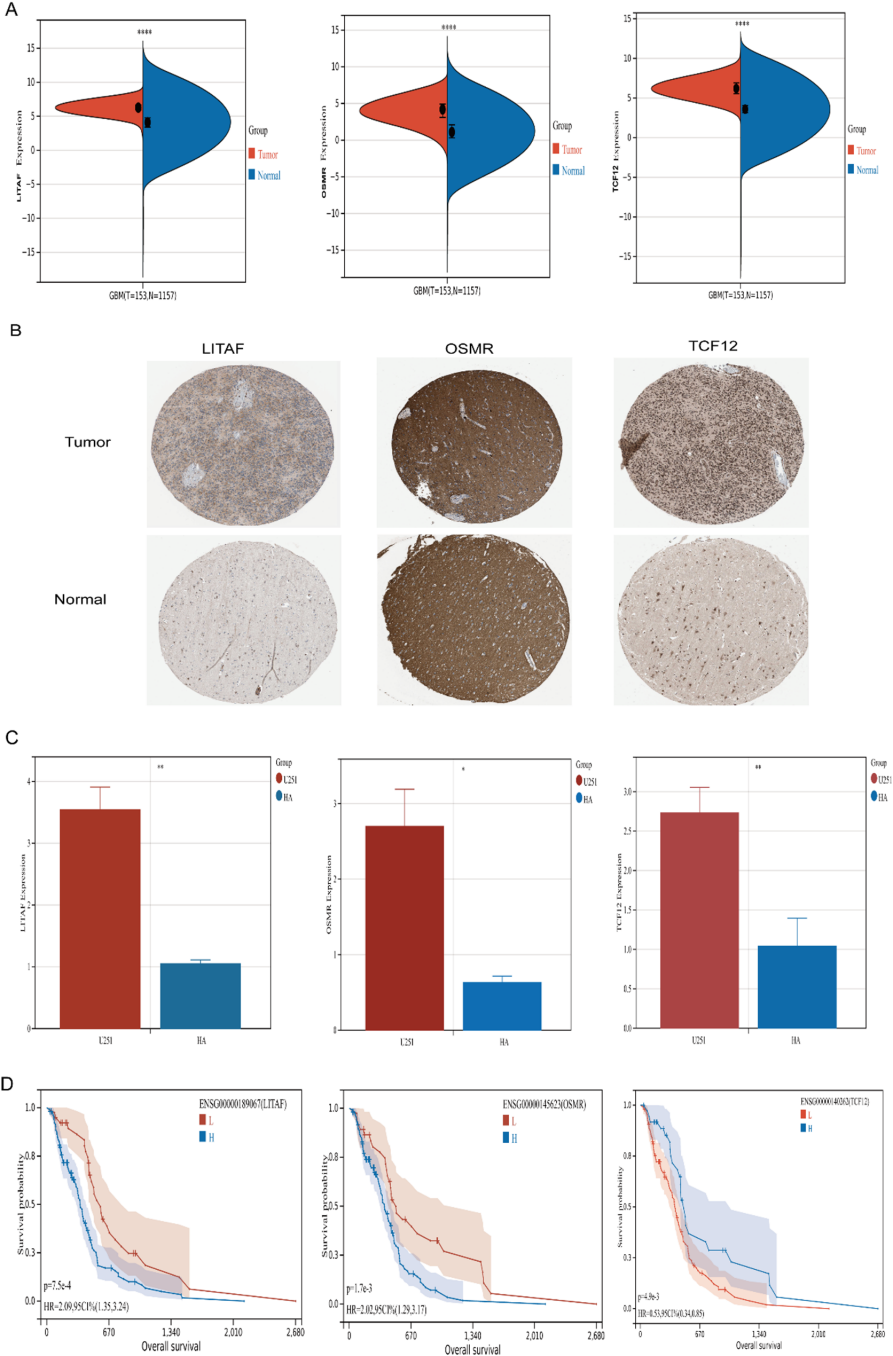
Analysis of signature genes expression. (**A**) The mRNA expression of 3 signature genes. (**B**) Immunohistochemistry images of LITAF https://www.proteinatlas.org/ENSG00000189067-LITAF/cancer/glioma#img; https://www.proteinatlas.org/ENSG00000189067-LITAF/tissue/cerebral+cortex#img ), OSMR (https://www.proteinatlas.org/ENSG00000145623-OSMR/cancer/glioma#img; https://www.proteinatlas.org/ENSG00000145623-OSMR/tissue/cerebral+cortex#img), and TCF12 (https://www.proteinatlas.org/ENSG00000140262-TCF12/cancer/glioma#img; https://www.proteinatlas.org/ENSG00000140262-TCF12/tissue/cerebral+cortex#img) in both normal cerebral cortex tissue and glioma tissue sourced from the Human Protein Atlas (HPA) database (https://www.proteinatlas.org/)(scale bar = 200 μm).(**C**) Relative mRNA levels of LITAF, OSMR, and TCF12 in U251 cells (*n* = 3) were significantly higher than in HA cells (*n* = 3). (**D**) Kaplan-Meier (K-M) survival curves comparing patients with high vs. low gene expression (*P < 0.05; **P < 0.01; ***P < 0.001; ****P < 0.0001)

After our search, database interrogation revealed protein expression data for three signature genes in the HPA. Comparative analysis demonstrated elevated *LITAF*,* OSMR*,* and TCF12* expression in glioma versus normal tissues at transcriptional level (Fig. 8A). Immunohistochemical validation showed concordant protein expression patterns for LITAF, OSMR, and TCF12 (Fig. 8B). The qRT-PCR results showed that relative mRNA levels of *LITAF*, *OSMR*, and *TCF12* in U251 cells (*n* = 3) were significantly higher than in HA cells (*n* = 3) (Fig. 8C). This expression concordance between transcriptomic and proteomic levels supports the prognostic utility of these biomarkers. To verify the impact of three genes on the prognosis of glioblastoma patients, we analyzed the TCGA-GBM database and found that high expression of *LITAF* and *OSMR* significantly shortened patient overall survival, while high expression of *TCF12* significantly extended patient overall survival. This further confirms that *TCF12* is a protective factor, whereas *LITAF* and *OSMR* are risk factors (Fig. 8D).

## Discussion

Emerging evidence suggests that bidirectional interactions between tumor cells and stromal elements critically influence tumor progression [5]. As confirmed contributors to tumor proliferation, angiogenesis, metastasis, and chemoresistance through paracrine signaling in the TME [24], CAFs warrant detailed investigation. In this study, we systematically characterized CAF heterogeneity in GBM using scRNA-seq, identifying five distinct CAF clusters with unique functional properties that differentially regulate TME biology. Supporting previous findings in hepatocellular carcinoma regarding CAF-secreted factors and related gene signatures as prognostic indicators [25], our analysis revealed three clusters significantly associated with GBM patient outcomes based on DEG-derived cluster scores. Pathway analysis revealed differential activation patterns across clusters in five key pathways: TP53 (cluster-specific p53 pathway alterations impacting invasion, apoptosis evasion, and stemness [26]), RTK-RAS (therapy resistance-linked transcriptional dysregulation [27]), Hippo (glioma progression and chemoresistance mechanisms [28]), TGF-β (calcium-mediated microenvironment communication [29]), and PI3K/AKT/mTOR (metabolic reprogramming associated with poor prognosis [30]). These pathway variations likely underlie the observed prognostic differences among CAF clusters.

Leveraging prognostic insights from three CAF clusters, we established a 3-gene signature (*LITAF*,* OSMR*,* TCF12*). While no significant mutational co-occurrence was observed between *LITAF* and *TCF12*, *OSMR* exhibited significant co-mutation with TP53 (*P* < 0.05). Missense SNVs may alter protein function, potentially contributing to gliomagenesis [31]. Although direct evidence linking these SNVs to GBM progression remains limited, our data suggest their putative regulatory roles. The 3 target genes are mainly distributed in the myofibroblastic CAF (myCAF) subpopulations, which propels GBM progression via ECM remodeling, immunosuppression, and treatment resistance. Targeting genes such as *LITAF*,* OSMR*, and *TCF12* could selectively disrupt myCAF-driven malignancy while preserving anti-tumor stromal functions. Functionally, OSMR - a leukocyte interleukin-6 receptor- activates JAK-STAT3 signaling upon binding oncostatin M (OSM), thereby driving glioma proliferation, invasion, and apoptosis resistance [32]. STAT3 activation upregulates matrix-remodeling enzymes (e.g., LOX/MMP) to promote collagen crosslinking and stromal desmoplasia, while inducing VEGF-mediated angiogenesis [33]. Additionally, the OSMR-STAT3 axis facilitates M2 macrophage polarization, PD-L1 expression, and T-cell suppression, collectively fostering immune evasion. Notably, OSMR-TP53 co-mutation may synergistically enhance malignancy through a STAT3-mutant p53 feedback loop, promoting epithelial-mesenchymal transition (EMT), matrix stiffening, and immune escape. Clinically, this molecular subtype could enable noninvasive prognosis stratification. Therapeutically, combined JAK-STAT3 and p53 pathway targeting may overcome treatment resistance. Pathway enrichment analysis linked the three signature genes to 31 oncogenic processes, prominently including IL6-JAK-STAT3 signaling, P53 pathway, and inflammatory response (Fig. 5A, B). This aligns with prior reports: JAK2/STAT3 inhibition (e.g., AG490) suppresses EGFRvIII-driven invasion in GBM [34], while VEGF-A blockade counteracts myeloid-derived suppressor cell (MDSC) differentiation [35]. These associations highlight promising mechanistic avenues for targeting CAF-mediated TME remodeling.

Emerging evidence highlights that CAF interactions with the TIME facilitate tumor progression [36]. Our analysis revealed two prognostic genes exhibiting significant positive correlations with immune scores, whereas a risk-associated gene demonstrated negative correlation. These findings suggest potential cross-regulation between these genetic markers and TIME components in GBM, indicating their therapeutic targeting potential. The TIME comprises diverse immune cell populations within tumor niches that collectively shape anti-tumor immunological responses. The previous researchers have developed an analytical tool termed “immuno-oncology biological research (IOBR)” to decipher the TME and its role in anti-tumor immunity. IOBR facilitates comprehensive characterization of TME features, immune interactions, and their impact on immunotherapy outcomes, enabling biomarker discovery and advancing precision medicine [37]. CAFs can establish immunosuppressive networks through crosstalk with these immune populations, enabling tumor immune evasion [38]. Intriguingly, our risk signature analysis identified inverse associations between predictive genes and many T cell subsets. Given the established role of T cells in tumor progression and the clinical validation of T cell-based immunotherapies (including checkpoint inhibitors and CAR-T therapies) [39], these findings warrant further investigation into the functional relationships between identified genes and adaptive immunity.

Nevertheless, Most patients exhibit intrinsic or adaptive resistance to immunotherapeutic interventions [40]. Our analysis demonstrated that the risk signature could stratify patients based on their likelihood of benefiting from immunotherapeutic interventions. Previous studies identified CAF-expressed endosialin as a regulator of macrophage recruitment and polarization in GBM [41]. Notably, our signature revealed positive correlations between risk-associated genetic factors and M2 macrophages, suggesting their involvement in macrophage polarization dynamics. Concurrently, our data established the CAF-derived signature as a predictor of anti-PD-L1 immunotherapy responsiveness. Our signature revealed positive correlations between risk-associated genetic factors and neutrophils, and mechanistic studies reveal CAF-mediated regulation of neutrophil survival, activation, and effector functions in GBM via the IL6-STAT3-PD-L1 signaling cascade [42], which may underlie this observation. Interestingly, we discover that some researchers have devised a comprehensive scoring system, “Integrated Machine Learning and Genetic Algorithm-driven Multimodal Analysis” (iMLGAM), which demonstrated excellent predictive performance for immune checkpoint blockade (ICB) outcomes across multiple cohorts, which would provide us with new ideas in our future research [43]. These findings provide novel insights into CAF-mediated remodeling of the tumor niche and immune landscape within the TME. However, mechanistic investigations remain necessary to elucidate CAF-TIME communication networks and their therapeutic implications for GBM immunotherapy.

Several limitations warrant acknowledgment. Primarily, the CAF clustering framework and prognostic signature were derived from retrospective public datasets, requiring validation in prospective multicenter GBM cohorts. Secondly, while we focused on the prognostic utility of the CAF-based signature, future research should delineate its molecular mechanisms in GBM pathogenesis, including trajectory inference, cell-cell communication, transcriptional regulatory networks and in vitro co-culture experiments and in vivo studies, the above extensive research findings would strengthen the biological validation of their CAF signature and its specific relevance to GBM pathobiology. Thirdly, Since the research began before WHO CNS5 widespread adoption, although the classification of gliomas in the TCGA-GBMLGG and CGGA databases has undergone changes (WHO CNS5), the molecular markers of glioma discovered by TCGA (such as IDH mutations, 1p/19q co-deletion, etc.) have been adopted by the WHO CNS5 as the classification criteria. We ensured the reliability of molecular typing through the following measures: using the dual indicators of IDH mutation and 1p/19q co-deletion to define the core subtype (consistent with the core principles of WHO CNS5). Finally, while this study establishes the clinical relevance of CAF-derived signatures, further investigation into the transcriptional regulators governing CAF heterogeneity is warranted. Future work will employ regulatory network inference (e.g., pySCENIC) to identify master transcription factors driving prognostically distinct CAF subtypes and the expression of signature genes, potentially revealing novel therapeutic targets for stroma-focused interventions in GBM.

## Conclusion

In summary, this study provides evidence for molecular heterogeneity among CAFs in GBM, suggesting five subpopulations with distinct profiles. Enrichment analysis of DEGs across these subgroups identified significant involvement in Antigen processing and presentation, ECM-receptor interaction, Vascular smooth muscle contraction, Cytoskeleton in muscle cells, PI3K-AKT signaling, and focal adhesion. Based on three prognosis-critical clusters, we propose a CAF-derived 3-gene risk signature (*LITAF/OSMR/TCF12*) that showed correlations with GBM outcomes in our cohort. Exploratory analyses revealed connections between this signature and immune microenvironment features, along with preliminary evidence for its potential utility in predicting responses to PD-L1 blockade. Furthermore, we constructed a nomogram integrating this signature with clinical parameters, which demonstrated improved performance in prognostic stratification compared to conventional factors in this dataset.

## Supplementary Information

Below is the link to the electronic supplementary material.

Supplementary Material 1

Supplementary Material 2

Supplementary Material 3

Supplementary Material 4

Supplementary Material 5

Supplementary Material 6

Supplementary Material 7

## Data Availability

The original contributions presented in the study are included in the article. Further inquiries can be directed to the corresponding authors.
