# Supplementary material for: Integrated single-cell and bulk transcriptomic profiling reveals cancer-associated fibroblast heterogeneity in glioblastoma and establishes a clinically actionable prognostic model and preliminary experimental validation
Source: Hereditas. 2025 Aug 26;162:173. doi: 10.1186/s41065-025-00548-8 (PMC12382188; doi:10.1186/s41065-025-00548-8)
Supplement: Supplementary file 1 — Supplementary Material 1 [file 41065_2025_548_MOESM1_ESM.docx]

**Supplementary Figure 1**. Quality control analysis of GBM scRNA-seq data processing. (A) Correlations between mitochondrial gene content and UMI/mRNA counts; (B) Pre-filtering distributions of mRNA/UMI counts with mitochondrial/rRNA content across samples; (C) Post-filtering distributions of mRNA/UMI counts with mitochondrial/rRNA content; (D) PCA visualization showing sample distribution and anchoring points.

**Supplementary Figure 2**. Clustering analysis and dimensionality reduction of cancer-associated fibroblast (CAF) populations. (A) Distribution of cellular subpopulations following unsupervised clustering analysis of all cells; (B) t-SNE projection visualizing expression patterns of canonical fibroblast marker genes; (C) Subpopulation distribution after secondary clustering of the fibroblast compartment; (D) t-SNE visualization of cluster-specific marker gene expression profiles across four CAF subpopulations

**Supplementary Figure 3.** t-SNE of high-quality cells to visualize cell clusters based on the expression of known marker genes.

**Supplementary Figure 4**. The expression of EPCAM across four CAF clusters.

**Supplementary Figure 5**. Mutation characteristics of risk signature genes**.** (A) Waterfall plot illustrating single nucleotide variant (SNV) mutations in four key genes. (B) Co-occurrence and mutual exclusivity patterns between key genes and the top 10 most mutated genes in tumors. (C)Copy number variation (CNV) status (gain, loss, or neutral) of the four key genes.

**Supplementary Figure 6**. Immune microenvironment associations of signature genes. (A) Stromal/immune/ESTIMATE score correlations with risk genes. (B) Immune score stratification by gene expression levels (Wilcoxon test). (C)CIBERSORT-derived immune cell infiltration correlations. (D) Differential infiltration of 22 immune cell subtypes between expression groups (Wilcoxon test). (**P* < 0.05; ***P* < 0.01; ****P* < 0.001; *****P* < 0.0001).
