# Supplementary figures and images for: Integrated single-cell and bulk transcriptomic profiling reveals cancer-associated fibroblast heterogeneity in glioblastoma and establishes a clinically actionable prognostic model and preliminary experimental validation
Source: Hereditas. 2025 Aug 26;162:173. doi: 10.1186/s41065-025-00548-8 (PMC12382188; doi:10.1186/s41065-025-00548-8)

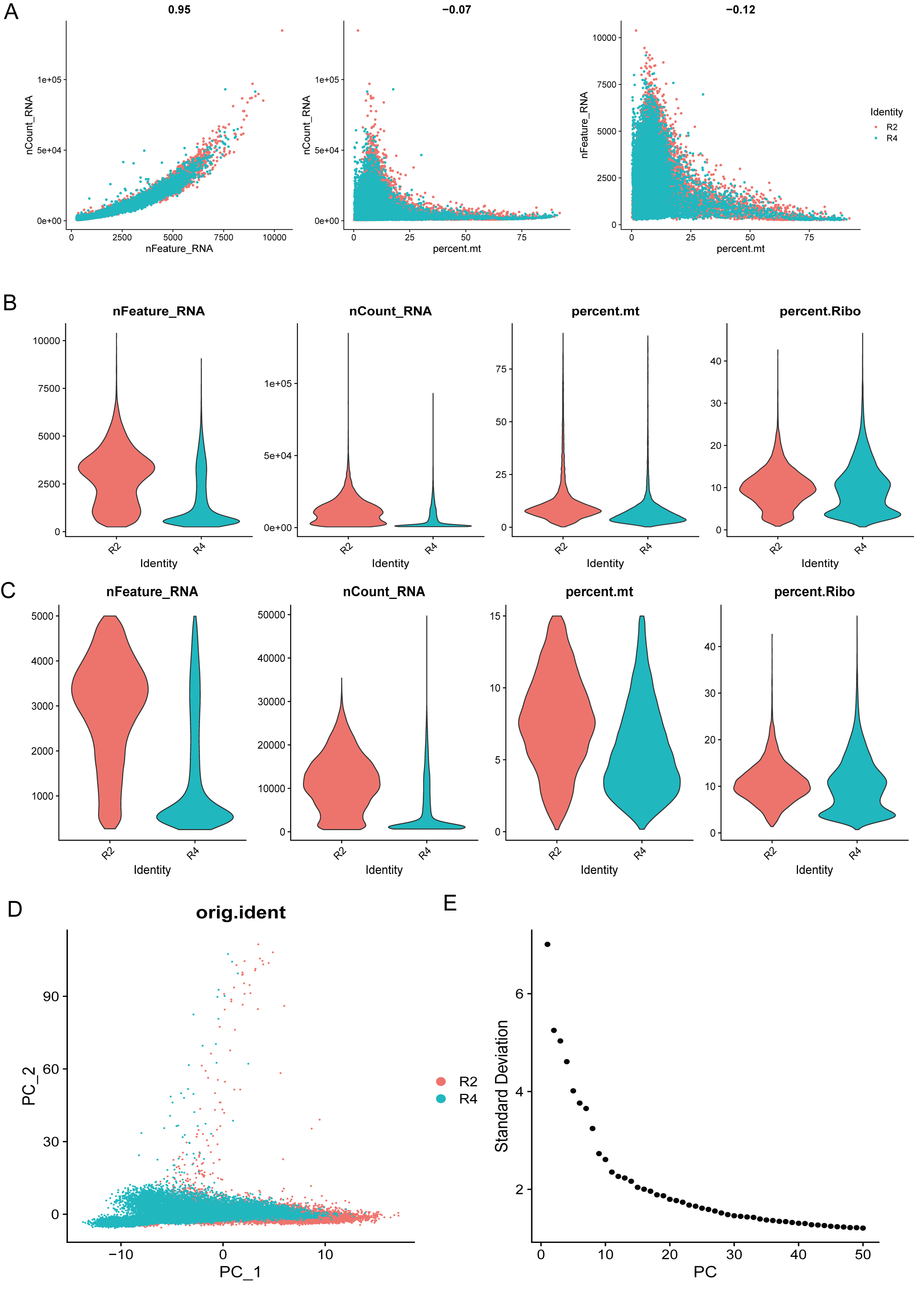

Supplement: Supplementary file 2 — Supplementary Material 2 [file 41065_2025_548_MOESM2_ESM.tif]

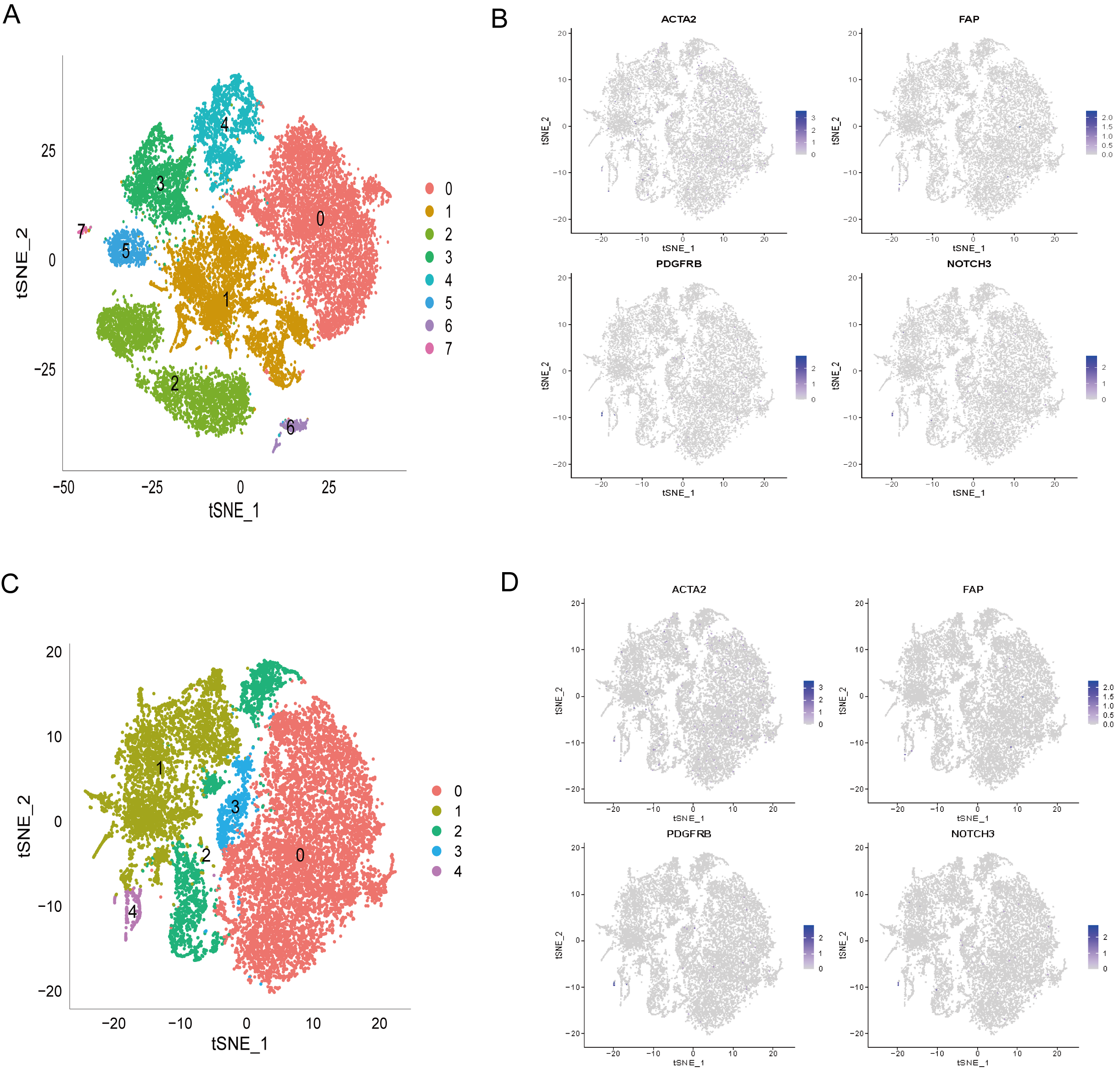

Supplement: Supplementary file 3 — Supplementary Material 3 [file 41065_2025_548_MOESM3_ESM.tif]

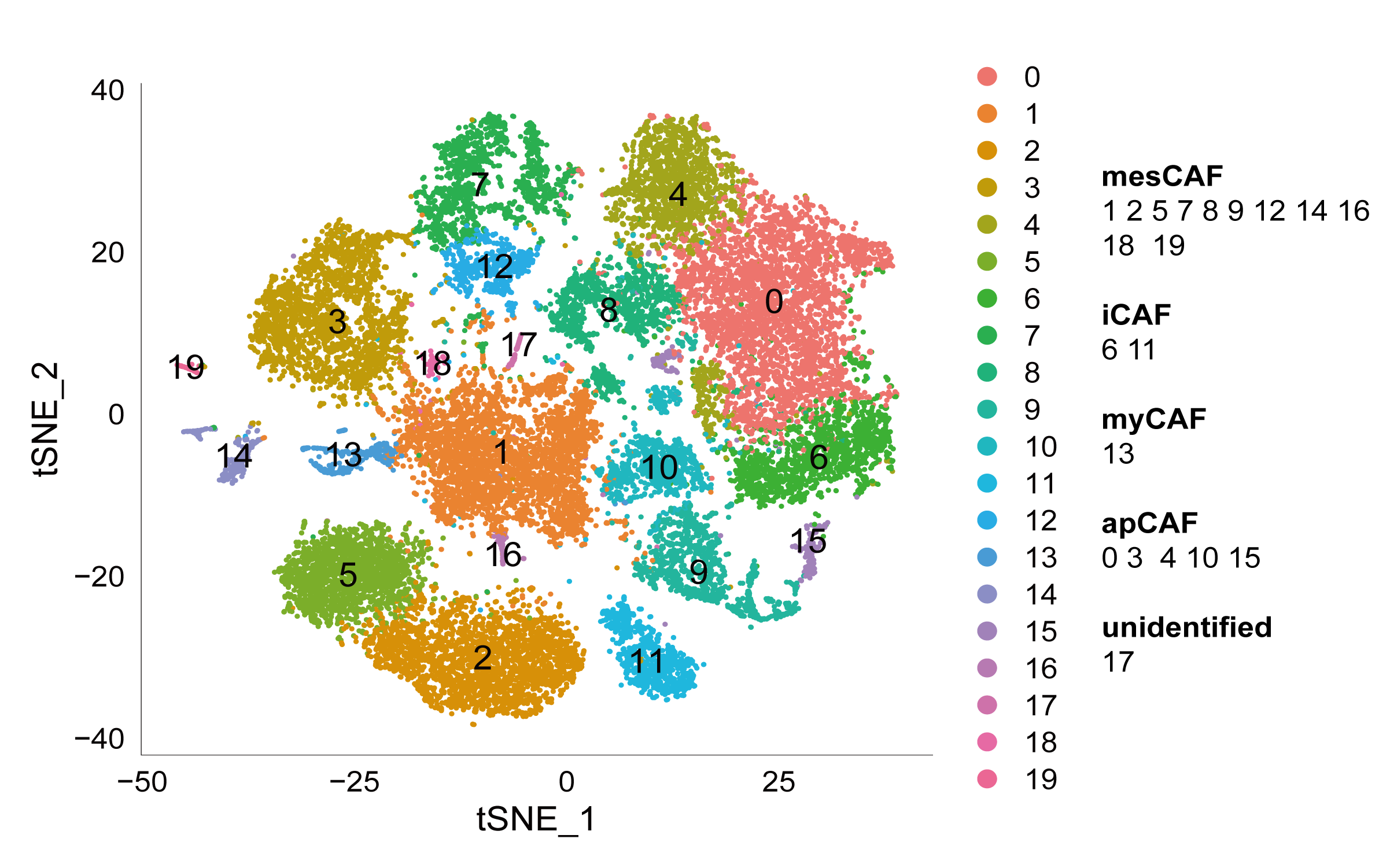

Supplement: Supplementary file 4 — Supplementary Material 4 [file 41065_2025_548_MOESM4_ESM.tif]

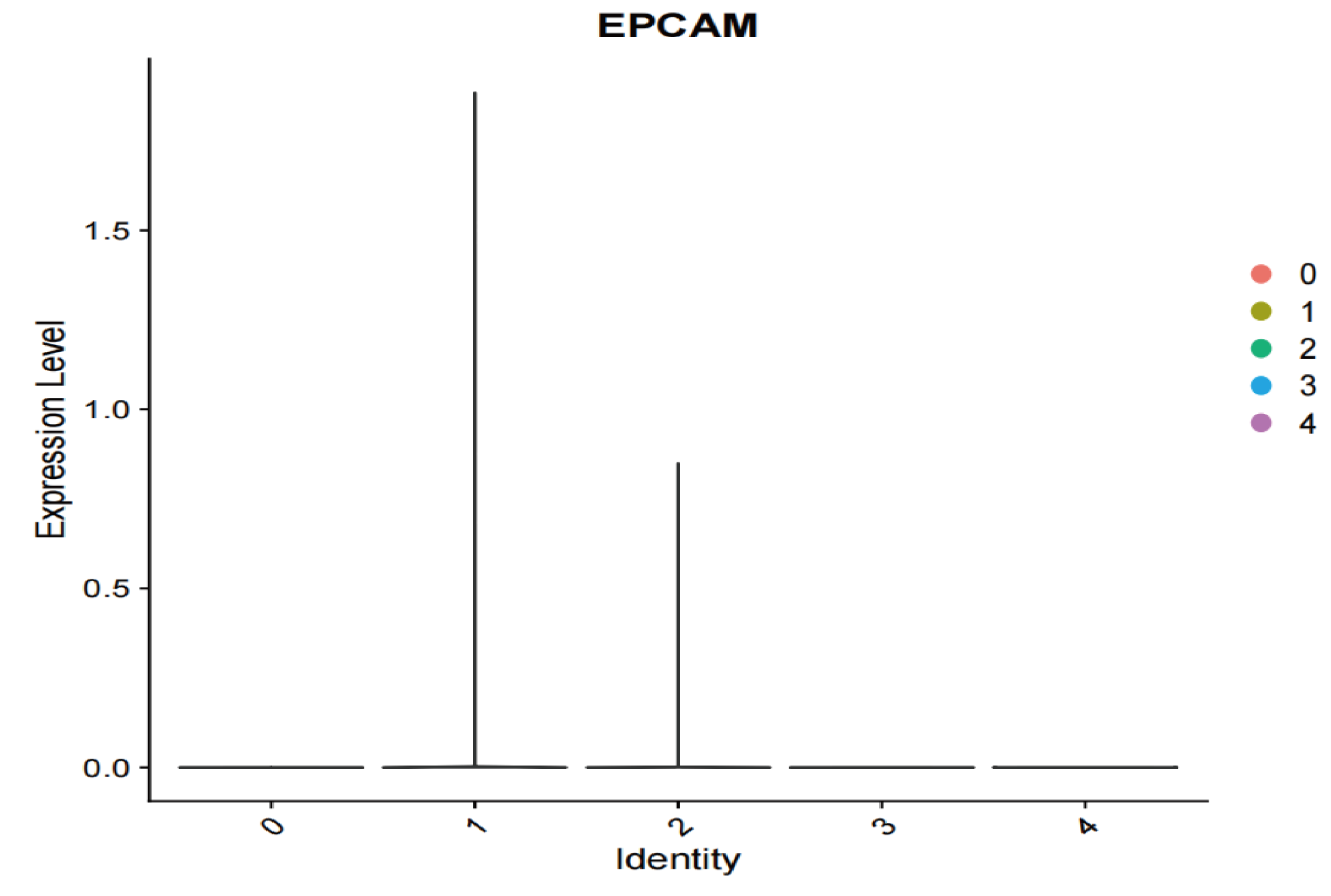

Supplement: Supplementary file 5 — Supplementary Material 5 [file 41065_2025_548_MOESM5_ESM.tif]

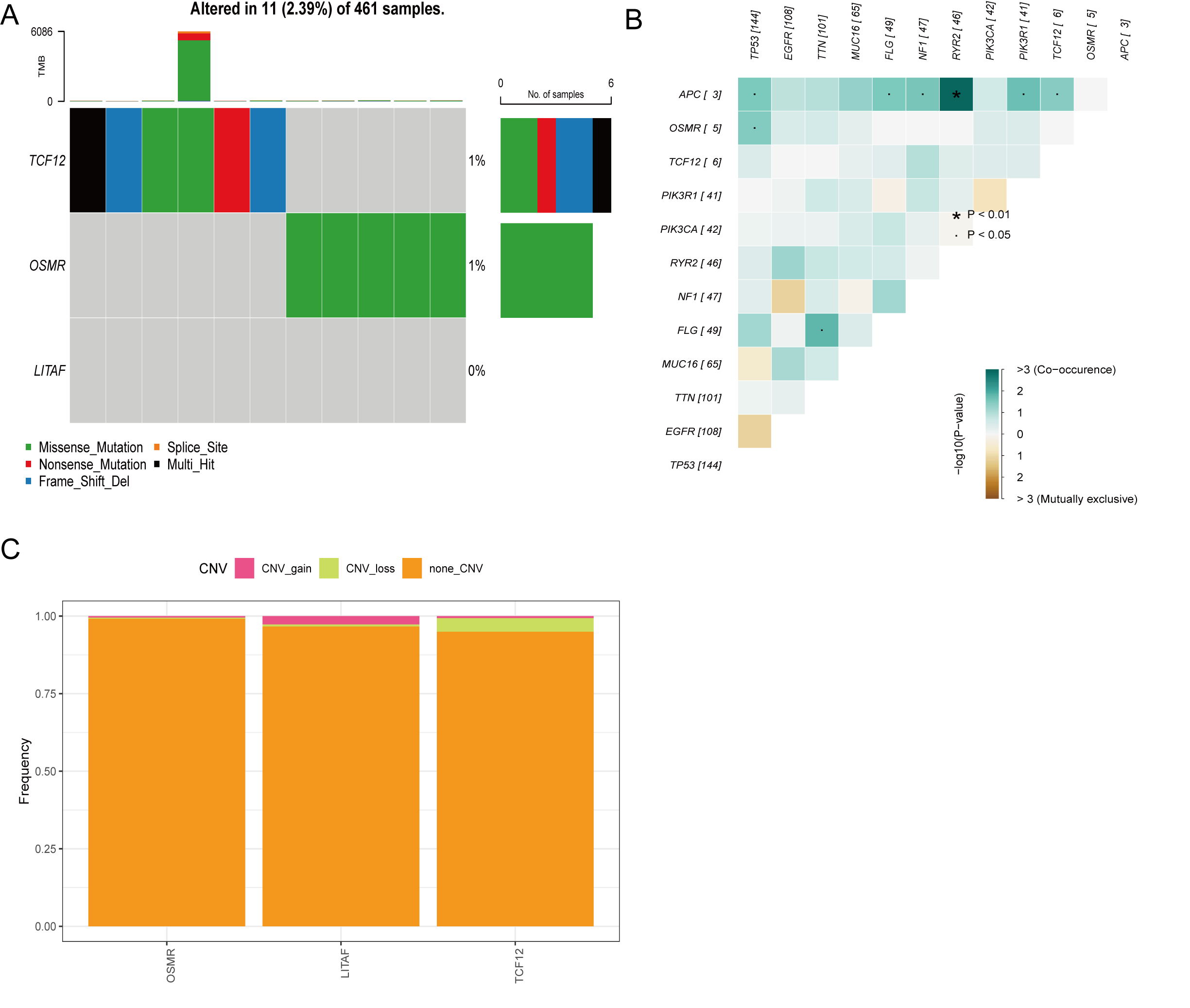

Supplement: Supplementary file 6 — Supplementary Material 6 [file 41065_2025_548_MOESM6_ESM.tif]

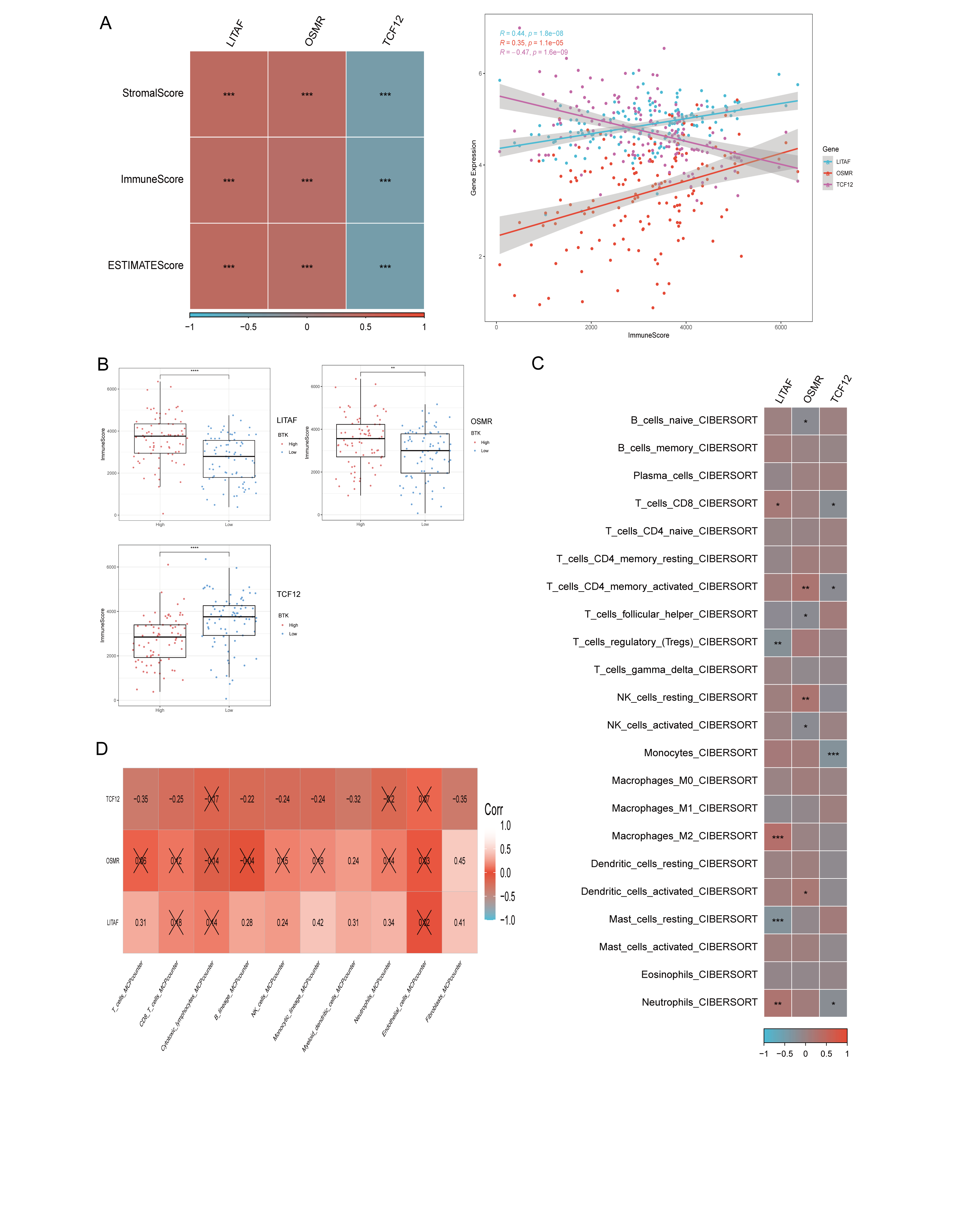

Supplement: Supplementary file 7 — Supplementary Material 7 [file 41065_2025_548_MOESM7_ESM.tif]
